# Study on geometry and morphology of proximal humerus in Northern Chinese population based on 3-D CT

**DOI:** 10.1186/s13018-023-03504-2

**Published:** 2023-01-17

**Authors:** Boyu Zhang, Haitao Guan, Zhipeng Ye, Yingze Zhang

**Affiliations:** 1 The School of Medicine, Nankai University, Tianjin, China; 2 Department of Orthopaedic Surgery, The Third Hospital of Hebei Medical University, Shijiazhuang, 050051 Hebei China; 3 Key Laboratory of Biomechanics of Hebei Province, Orthopaedic Research Institution of Hebei Province, Shijiazhuang, 050051 Hebei People’s Republic of China; 4 NHC Key Laboratory of Intelligent Orthopeadic Equipment, Third Hospital of Hebei Medical University, Shijiazhuang, Hebei People’s Republic of China; 5 Chinese Academy of Engineering, Beijing, People’s Republic of China

**Keywords:** Humeral morphology, Humeral geometry, Shoulder arthroplasty, Three-dimensional analysis, Computed tomography

## Abstract

**Background:**

This study investigated the characteristics of humeral geometric and morphological parameters in northern Chinese population by three-dimensional measurements, and compared whether there were differences in humeral morphology among populations from different geographical regions.

**Methods:**

Computed tomography scans of 80 humerus were obtained, reconstructed and measured. Differences in humeral morphological parameters between genders and sides were compared. Correlation analysis was used to explore possible correlations among the parameters. The differences in humeral geometric morphometric parameters between Western and East Asian populations were compared according to pool results of present and previous studies.

**Results:**

The average (and standard deviation) of humeral head radius curvature, arc angle, diameter, and thickness was 151.79 ± 6.69°, 23.36 ± 2.08 mm, 44.83 ± 3.92 mm and 17.55 ± 1.84 mm in coronal humeral head plane, and 152.05 ± 8.82°, 21.81 ± 1.88 mm, 41.77 ± 3.44 mm and 16.52 ± 1.92 mm in transversal humeral head plane. The average of the humeral head medial offset and posterior offset was 7.34 ± 2.47 mm and 0.08 ± 1.72 mm. Humeral head inclination angle, arc angle and radius curvature of humeral neck-shaft averaged 137.69 ± 4.92°, 34.7 ± 5.29° and 55.76 ± 13.43 mm. Superior, inferior, anterior, posterior concave angle of humeral anatomical neck averaged 150.41 ± 10.91°, 146.55 ± 10.12°, 146.43 ± 13.53° and 149.33 ± 14.07°. The average of height of the greater tuberosity, height of the lesser tuberosity, depth, concave angle and volume of the intertubercular groove was 14.19 ± 1.7 mm, 8.9 ± 1.54 mm, 0.92 ± 0.31 mm3, 31.28 ± 9.61 mm, 4.98 ± 1.19 mm and 89.35 ± 17.62°. The upper angle of the greater tuberosity averaged 161.04 ± 7.84°, the upper angle of the greater tuberosity was 165.94 ± 3.6°. Differences in parameters of proximal humerus between genders and sides were found. There was no correlation between parameters of proximal humerus and age. Correlations were found among humeral morphological parameters. East Asian populations differed in proximal humeral morphology from Western populations.

**Conclusions:**

This study will provide references for diagnosing and classifying shoulder disease, designing prosthesis and instrument, enhancing surgical precision and guiding patient recovery.

## Background

The proximal humerus has an important role in daily life as part of the shoulder joint. Diseases that occurred in proximal humerus such as rotator cuff tears and proximal humeral fractures are common in clinic and have gradually increased incidence in recent years, bringing pain and financial burden to patients [1, 2]. Detailed understanding about the morphology of humerus is the theoretical foundation that could essentially improve the diagnosis and treatment quality of surgeons. Furthermore, previous studies show changes in skeletal morphology with aging, including femoral and spine [3, 4], study on humeral morphology could verify whether this phenomenon occurred at upper limb.

Investigators have used a variety of methods such as cadaveric measurements [5] and X-ray measurements [6] to measure proximal humeral morphology to optimize shoulder prosthesis design and improve treatment outcomes for the shoulder disease.

Skeletal morphology was measured more precisely on multi-plane and multi-visual angle due to the increasing capacity of computed tomography techniques as well as computed three-dimensional (3D) reconstruction [7]. In this study, computed tomography (CT) images of the proximal humerus collected from a cohort of northern Chinese subjects were reconstructed and measured so that we can understand the proximal humerus morphology in northern Chinese population, analyze correlation between skeletal morphology and other parameters including age and gender. We summarized the results of previous studies on East Asian populations and compared with Western populations. The aim of this study was to provide accurate reference data for the anatomical morphology of proximal humerus and identify difference of the humeral morphology among different human species.

## Method and materials

The research was approved by ethics committee of our hospital (2020-014-1) and conducted in accordance with the Declaration of Helsinki. CT image data of humerus which were taken in the Third Hospital of Hebei Medical University from 2019 to 2021 have been included in this study. Image data were obtained in Digital Imaging and Communication in Medicine (DICOM) data format. CT images were all scanned on a Siemens 64 row spiral CT scanner by professionals. The scanning and reconstruction slice thickness were both ≤ 1 mm. Exclusion criteria included: 1. Incomplete baseline data, 2. Suboptimal imaging quality, 3. Fractures, bone defects, bone disease, bone tumors in the middle and upper humerus and 4. Severe osteoporosis or autoimmune diseases.

**Fig. 1:**
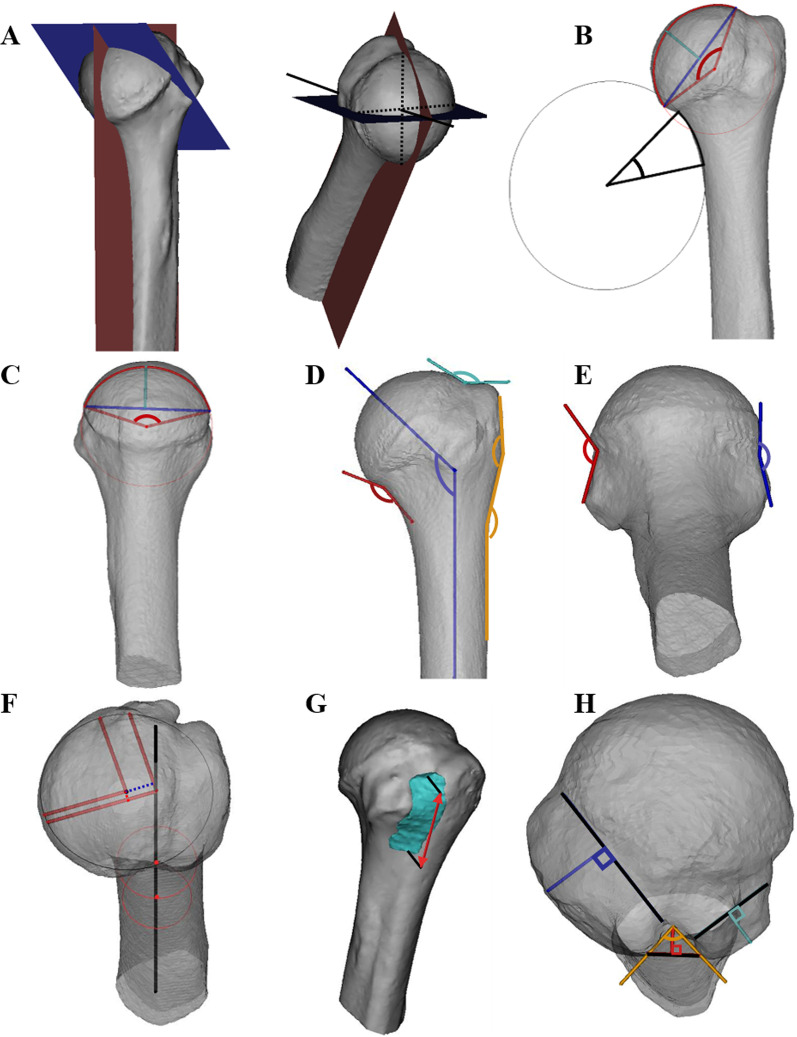
The measured parameters of the humerus and the measuring method. **A** The red plane indicated cHHP. The cHHP was defined by two intersecting lines, the proximal humeral shaft axis and the line which pass through the center of the biggest osculating circle of humeral head in transverse plane which is perpendicular to proximal humeral shaft axis and perpendicular to the longest wiring of the anterior and posterior margins of the humeral head in transverse plane. The blue plane indicated tHHP. The tHHP was perpendicular to the cHHP and that contained the humeral head axis which pass through the center of the osculating circle of humeral head in cHHP and perpendicular to the wiring of the superior and inferior margins of the humeral head in cHHP; **B** the radius of the red circle indicated RCHH in cHHP, the red angle indicated AAHH in cHHP, the blue line indicated DHH in cHHP, the cyan line indicated THH in cHHP, the radius of the black circle indicated RCNS, and the black angle indicated AANS; **C** the radius of the red circle indicated RCHH in tHHP, the red angle indicated AAHH in tHHP, the blue line indicated DHH in tHHP, and the cyan line indicated THH in tHHP; **D** the blue angle indicated IA which is defined as the included angle of the humeral head axis and the humeral shaft axis, the red angle referred to the inferior CAHAN, the cyan angle referred to the superior CAHAN, the upper orange angle referred to UAGT, the lower orange angle referred to LAGT; **E** the red angle referred to the anterior CAHAN, and the blue angle referred to the posterior CAHAN; **F** the red dotted line referred to PO, and the blue dotted line referred MO; **G** the cyan region referred to VIG, and the red line referred to LIG; **H** the blue line referred to HGT, the cyan line referred to HLT, the red line referred to HIG, and the orange angle referred to CAIG

CT image processing and three-dimensional modeling were performed using Mimics software (Materialise, Leuven, Belgium). Influence of patient posture on CT imaging was eliminated by realignment of the examination plane. The 3D humerus model was reconstructed according to CT thresholds and measurements were performed with the assistance of two-dimensional (2D) images and 3D models. Important anatomical geometry parameters of the proximal humerus were measured, including humeral head radius curvature (RCHH), arc angle (AAHH), diameter (DHH), thickness (THH) in coronal humeral head plane (cHHP) and transversal humeral head plane (tHHP), humeral head inclination angle (IA), arc angle (AANS) and radius curvature (RCNS) of the humeral neck-shaft, humeral head medial offset (MO), posterior offset (PO), superior, inferior, anterior, posterior concave angle of humeral anatomical neck (CAHAN), height of the greater tuberosity (HGT), height of the lesser tuberosity (HLT), depth (DIG), concave angle (CAIG) and volume (VIG) of the intertubercular groove, the upper angle of the greater tuberosity (UAGT) and the lower angle of the greater tuberosity (LAGT). The vertical axis was adjusted to the proximal humeral shaft axis which was the axis that passes through the middle of the metaphyseal cylinder [8]. The anatomical neck was defined as the concave surrounded by the landmarks [9]. The measured methods and parameters are shown specifically in Fig. 1.

SPSS26 (SPSS Inc, Armonk, NY) was used in statistical analysis of data. Individual parameters were described in terms of mean value and standard deviation. All parameters were tested for normality using the K-S test. Independent sample t tests were used to compare gender and side difference for normally distributed continuous variables, and Kruskal–Wallis tests for non-normally distributed continuous variables. Pearson correlation analysis was used for normally distributed continuous variables to explore possible correlations among parameters, Spearman correlation analysis for non-normally distributed continuous variables. *P* value < 0.05 was considered statistically significant.

**Fig. 2:**
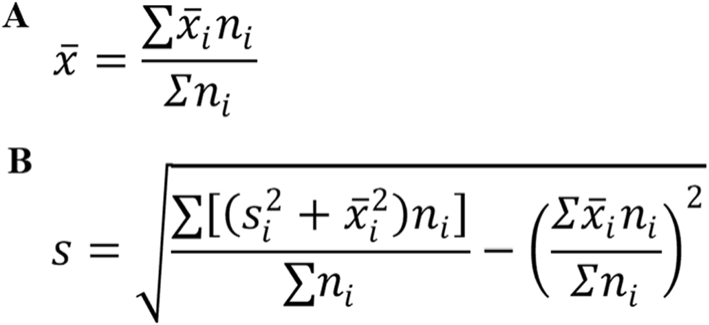
Methods for pooling statistical parameters across multiple studies. **A** The formula which pooled the average of multiple studies into one; **B** the formula which pooled the standard deviation of multiple studies into one

We performed a pooled calculation of mean and standard deviation from previous studies using the formula shown in Fig. 2. The parameters from Western population [5–7, 10–17] and East Asian population [8, 11, 18, 19] were contrasted by summary data *T* test. Similarly, *P* value < 0.05 was considered statistically significant.

## Results

CT scans of 80 humeral (38 left and 42 right, 42 males and 38 females) from objects, who were a mean age of 46.47 ± 13.14, were included in this study. Data characteristics of all tested parameters are shown in Table 1.

**Table 1:** Morphological parameters

	1/4 quartile 1/4	Average	3/4 quartile
Age	33.75	48.15 ± 15.32	60.00
*Position parameters of humeral head*			
MO (mm)	5.80	7.34 ± 2.47	9.23
PO (mm)	− 1.09	0.08 ± 1.72	1.39
*Dimension parameters of humeral head*			
AAHH in cHHP (°)	146.84	151.79 ± 6.69	156.89
RCHH in cHHP(mm)	21.66	23.36 ± 2.08	24.96
DHH in cHHP (mm)	41.57	44.83 ± 3.92	48.22
THH in cHHP (mm)	16.37	17.55 ± 1.84	19.04
AAHH in tHHP (°)	145.8	152.05 ± 8.82	157.36
RCHH in tHHP (mm)	20.02	21.81 ± 1.88	23.04
DHH in tHHP (mm)	39.36	41.77 ± 3.44	44.62
THH in tHHP (mm)	15.11	16.52 ± 1.92	17.92
*Parameters of metaphysis*			
IA (°)	134.11	137.69 ± 4.92	141.72
AANS (°)	31.2	34.7 ± 5.29	37.53
RCNS (mm)	44.5	55.76 ± 13.43	63.45
*Parameters of anatomical neck*			
Superior CAHAN (°)	143.62	150.41 ± 10.91	157.04
Inferior CAHAN (°)	139.03	146.55 ± 10.12	151.59
Anterior CAHAN (°)	142.30	146.43 ± 13.53	154.05
Posterior CAHAN (°)	144.39	149.33 ± 14.07	157.34
*Parameters of greater, less tuberosity and intertubercular groove*			
HGT (mm)	12.95	14.19 ± 1.7	15.48
HLT (mm)	7.88	8.9 ± 1.54	9.75
VIG (mm^3^)	0.68	0.92 ± 0.31	1.08
LIG (mm)	24.33	31.28 ± 9.61	34.91
DIG (mm)	4.4	4.98 ± 1.19	5.39
CAIG (°)	77.07	89.35 ± 17.62	102.28
UAGT (°)	157.35	161.04 ± 7.84	165.83
LAGT (°)	163.89	165.94 ± 3.6	168.44

### Parameters of humeral head

In our cohort, the mean AAHH, RCHH, DHH and THH in cHHP were 151.79 ± 6.69°, 23.36 ± 2.08 mm, 44.83 ± 3.92 mm and 17.55 ± 1.84 mm. AAHH, RCHH, DHH and THH in tHHP were 152.05 ± 8.82°, 21.81 ± 1.88 mm, 41.77 ± 3.44 mm and 16.52 ± 1.92 mm. The mean value of MO and PO which could describe the relative position of the humeral head to the proximal humeral shaft was 7.34 ± 2.47 mm and 0.08 ± 1.72 mm.

### Parameters of anatomical neck

Superior CAHAN averaged 150.41 ± 10.91°; inferior CAHAN averaged 146.55 ± 10.12°; anterior CAHAN averaged 146.43 ± 13.53°; posterior CAHAN averaged 149.33 ± 14.07°.

### Parameters of metaphysis

The mean IA of included subjects was 137.69 ± 4.92°, the mean AANS was 34.7 ± 5.29°, and the mean RCNS was 55.76 ± 13.43 mm.

### Parameters of greater, less tuberosity and intertubercular groove

On average, HGT was 14.19 ± 1.7 mm, HLT was 8.9 ± 1.54 mm, VIG, LIG, DIG and CAIG was 0.92 ± 0.31 mm^3^, 31.28 ± 9.61 mm, 4.98 ± 1.19 mm and 89.35 ± 17.62°. UAGT averaged 161.04 ± 7.84°, LAGT was 165.94 ± 3.6°.

### Difference according to gender and side

The comparative results are shown in Table 2. Compared with women, RCHH (*P* < 0.001*), DHH (*P* < 0.001*), THH (*P* < 0.001*) in cHHP and tHHP, as well as AAHH in tHHP (*P* < 0.001*) are greater in men. Similar to the results of Noboru Matsumura [18], there was no difference by sex in PO (*P* = 0.2) or MO (*P* = 0.256). There were no differences in superior (*P* = 0.453), inferior (*P* = 0.476), anterior (*P* = 0.268) CAHAN between two groups; however, posterior (*P* = 0.003*) CAHAN was larger in the male group. IA (*P* = 0.916), AANS (*P* = 0.086) and RCNS (*P* = 0.87) in male were statistically indistinguishable from those in female. Men had significantly HGT (*P* < 0.001*), HLT (*P* = 0.001*), VIG (*P* = 0.002*) and DIG (*P* = 0.001*) than women, LIG (*P* = 0.108) and CAIG (*P* = 0.461) did not differ between genders. UAGT (*P* = 0.661) and LAGT (*P* = 0.217) were 161.72 ± 6.08° and 165.7 ± 3.45° in male, 160.29 ± 9.28° and 166.24 ± 3.7° in female, indicating that the difference in the undulation shape of the lateral aspect of the greater tuberosity was not statistically significant between men and women. There was no difference in proximal humeral parameters between left and right sides except for lesser tuberosity height.

**Table 2:** Sex and side differences in morphological parameters

	Total	Male	Female	*P* value	Left	Right	*P* value
Age	48.15 ± 15.32	45.40 ± 15.86	51.18 ± 13.9	0.096	46.47 ± 12.97	49.67 ± 16.89	0.302
*Position parameters of humeral head*							
MO (mm)	7.34 ± 2.47	7.56 ± 2.41	7.1 ± 2.49	0.256	7.9 ± 2.47	6.84 ± 2.33	0.059
PO (mm)	0.08 ± 1.72	− 0.07 ± 1.76	0.24 ± 1.63	0.2	− 0.05 ± 1.75	0.19 ± 1.66	0.537
*Dimensional parameters of humeral head*							
AAHH in cHHP (°)	151.79 ± 6.69	151.28 ± 6.19	152.35 ± 7.08	0.484	152.75 ± 6.42	150.92 ± 6.73	0.23
RCHH in cHHP (mm)	23.36 ± 2.08	24.93 ± 1.31	21.62 ± 1.23	< 0.001^*^	23.43 ± 2.09	23.29 ± 2.05	0.77
DHH in cHHP (mm)	44.83 ± 3.92	47.88 ± 2.24	41.46 ± 2.32	< 0.001^*^	45.04 ± 3.94	44.65 ± 3.86	0.668
THH in cHHP (mm)	17.55 ± 1.84	18.62 ± 1.39	16.37 ± 1.5	< 0.001^*^	17.72 ± 1.89	17.4 ± 1.75	0.449
AAHH in tHHP (°)	152.05 ± 8.82	152.36 ± 7.96	151.71 ± 9.57	< 0.001^*^	153.28 ± 9.64	150.94 ± 7.74	0.244
RCHH in tHHP (mm)	21.81 ± 1.88	23.28 ± 1.16	20.19 ± 1	< 0.001^*^	21.77 ± 1.88	21.85 ± 1.87	0.846
DHH in tHHP (mm)	41.77 ± 3.44	44.52 ± 1.89	38.74 ± 1.89	< 0.001^*^	41.67 ± 3.19	41.87 ± 3.61	0.807
THH in tHHP (mm)	16.52 ± 1.92	17.48 ± 1.45	15.46 ± 1.8	< 0.001^*^	16.51 ± 1.67	16.53 ± 2.1	0.981
*Parameters of metaphysis*							
IA (°)	137.69 ± 4.92	137.74 ± 4.64	137.62 ± 5.15	0.916	137.19 ± 5	138.13 ± 4.73	0.482
AANS (°)	34.7 ± 5.29	35.59 ± 4.98	33.72 ± 5.39	0.086	34.38 ± 5.83	34.99 ± 4.66	0.661
RCNS (mm)	55.76 ± 13.43	56.00 ± 12.69	55.5 ± 14.03	0.87	57.56 ± 13.6	54.13 ± 12.91	0.263
*Parameters of anatomical neck*							
Superior CAHAN (°)	150.41 ± 10.91	149.52 ± 10.54	151.39 ± 11.09	0.453	148.25 ± 11.39	152.36 ± 9.94	0.097
Inferior CAHAN (°)	146.55 ± 10.12	147.34 ± 9.64	145.69 ± 10.45	0.476	145.19 ± 9.92	147.78 ± 10.03	0.262
Anterior CAHAN (°)	146.43 ± 13.53	147.88 ± 13.55	145.17 ± 12.86	0.268	150.72 ± 14.06	143.52 ± 13.37	0.058
Posterior CAHAN (°)	149.33 ± 14.07	153.1 ± 13.92	144.84 ± 13.16	0.003^*^	149.66 ± 12.81	148.07 ± 13.79	0.268
*Parameters of greater, less tuberosity and intertubercular groove*							
HGT (mm)	14.19 ± 1.7	14.93 ± 1.49	13.38 ± 1.52	< 0.001^*^	13.98 ± 1.72	14.38 ± 1.64	0.304
HLT (mm)	8.9 ± 1.54	9.43 ± 1.66	8.31 ± 1.12	0.001^*^	9.29 ± 1.5	8.54 ± 1.47	0.031^*^
VIG (mm^3^)	0.92 ± 0.31	1.02 ± 0.29	0.81 ± 0.29	0.002^*^	0.94 ± 0.31	0.9 ± 0.3	0.572
LIG (mm)	31.28 ± 9.61	32.44 ± 9.29	30 ± 9.67	0.108	33.12 ± 10.63	29.62 ± 8.1	0.162
DIG (mm)	4.98 ± 1.19	5.34 ± 1.36	4.58 ± 0.78	0.001^*^	5.05 ± 1.05	4.92 ± 1.29	0.553
CAIG (°)	89.35 ± 17.62	87.94 ± 17.45	90.9 ± 17.47	0.461	87.24 ± 15.69	91.25 ± 18.83	0.184
UAGT (°)	161.04 ± 7.84	161.72 ± 6.08	160.29 ± 9.28	0.661	159.35 ± 9.08	162.57 ± 6.03	0.12
LAGT (°)	165.94 ± 3.6	165.66 ± 3.45	166.24 ± 3.7	0.217	165.76 ± 3.8	166.1 ± 3.36	0.677

*Significant at 0.05 level

### Correlation analysis of proximal humeral parameters

**Fig. 3:**
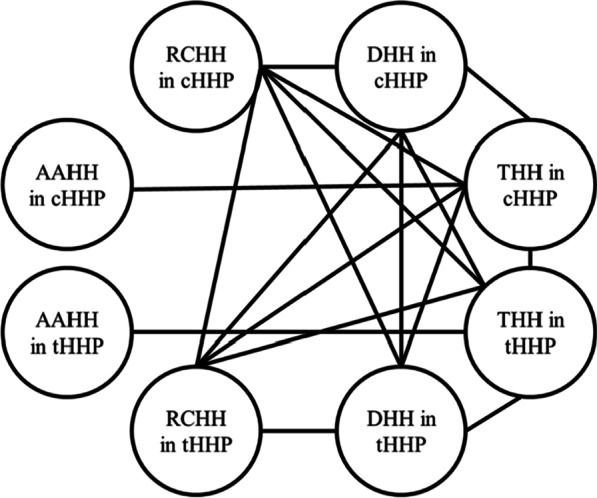
The mutual correlation between the dimensional parameters of humeral head

The results of the correlation analysis are shown in Tables 3, 4, 5, 6 and 7. There was none of the measured parameters showing significant correlation with age. A complex and extensive association was revealed among humeral head dimensional parameters. The correlation of parameters with each other within this dataset was concretely exhibited in Fig. 3. No correlation was found between humeral head position parameters. The results of correlation analysis among anatomical neck concavity angle parameters were negative as well. There was a correlation between the AANS and RCNS (*r *=  − 0.642, *p* < 0.001). There was a correlation between the VIG and LIG (*r* = 0.585, *p* < 0.001), DIG (*r* = 0.563, *p* < 0.001), respectively.

**Table 3:** Correlation between position parameters of humeral head

	Age	MO	PO
Age	1		
MO	− 0.044	1	
PO	0.153	− 0.108	1

**Table 4:** Correlation between dimensional parameters of humeral head

	Age	AAHH in cHHP	RCHH in cHHP	DHH in cHHP	THH in cHHP	AAHH in tHHP	RCHH in THHP	DHH in tHHP	THH in tHHP
Age	1								
AAHH in cHHP	− 0.051	1							
RCHH in cHHP	− 0.03	− 0.253*	1						
DHH in cHHP	− 0.066	− 0.078	0.968**	1					
THH in cHHP	− 0.07	0.533**	0.642**	0.763**	1				
AAHH in tHHP	− 0.054	0.214	0.032	0.1	0.138	1			
RCHH in THHP	− 0.015	− 0.12	0.893**	0.880**	0.666**	− 0.157	1		
DHH in tHHP	− 0.018	− 0.08	0.896**	0.913**	0.678**	0.128	0.930**	1	
THH in tHHP	− 0.072	0.096	0.603**	0.656**	0.589**	0.607**	0.528**	0.687**	1

*Significant at 0.05 level

**Significant at 0.01 level

Data with P < 0.05 and |r| > 0.5 in the table are marked in bold

**Table 5:** Correlation between parameters of metaphysis

	age	IA	AANS	RCNS
age	1			
IA	0.105	1		
AANS	0.001	− 0.205	1	
RCNS	− 0.008	0.004	− 0.642**	1

**Significant at 0.01 level

Data with P < 0.05 and |r| > 0.5 in the table are marked in bold

**Table 6:** Correlation between Parameters of anatomical neck

	Age	Superior CAHAN	Inferior CAHAN	Anterior CAHAN	Posterior CAHAN
Age	1				
Superior CAHAN	− 0.038	1			
Inferior CAHAN	− 0.098	− 0.114	1		
Anterior CAHAN	− 0.199	0.208	0.202	1	
Posterior CAHAN	− 0.15	− 0.085	0.220*	0.109	1

*Significant at 0.05 level

**Table 7:** Correlation between parameters of greater, less tuberosity and intertubercular groove

	Age	HGT	HLT	VIG	LIG	DIG	CAIG	UAGT	LAGT
Age	1								
HGT	− 0.163	1							
HLT	0.153	0.209	1						
VIG	0.049	0.256*	0.353**	1					
LIG	− 0.084	0.319**	0.222*	0.585**	1				
DIG	0.196	0.115	0.298**	0.563**	0.164	1			
CAIG	− 0.058	− 0.058	− 0.068	− 0.307**	− 0.410**	− 0.420**	1		
UAGT	0.248*	− 0.063	0.13	0.159	− 0.051	0.042	0.052	1	
LAGT	− 0.033	− 0.094	0.045	− 0.117	− 0.258*	− 0.004	0.211	0.183	1

*Significant at 0.05 level

**Significant at 0.01 level

Data with P < 0.05 and |r| > 0.5 in the table are marked in bold

### Comparison with other population

Compared with Western cohort, eastern Asian cohort have a smaller average value of IA (*P* < 0.001^*^), RCHH in cHHP (*P* < 0.001^*^), DHH in cHHP (*P* < 0.001^*^) and MO (P < 0.001^*^), and a larger average value of THH in cHHP (*P* < 0.032) and AAHH in cHHP (*P* < 0.001^*^). There was no difference in PO (*P* < 0.463). The comparative results are shown in Table 8.

**Table 8:** Comparison of geometric measurements among studies

Anatomical parameter	Western cohort		Eastern Asian cohort		
	Sample size	Mean ± SD	Sample size	Mean ± SD	*P* Value
Inclination angle (°)	1121	137.2 ± 5.5	558	134.1 ± 4.4	< 0.001^*^
Humeral head curvature on coronal humeral head plane (mm)	1179	25.2 ± 3.7	558	22.2 ± 3.4	< 0.001^*^
Humeral head thickness on coronal humeral head plane (mm)	487	16.7 ± 2.1	398	17.0 ± 1.9	0.032
Humeral head diameter on coronal humeral head plane (mm)	1037	49.1 ± 6.7	160	43.9 ± 3.9	< 0.001^*^
Humeral head arc on coronal humeral head plane (°)	313	147.7 ± 7.4	218	152.8 ± 6.1	< 0.001^*^
Medial offset (mm)	314	6.6 ± 2.0	500	6.0 ± 1.8	< 0.001^*^
Posterior offset (mm)	293	1.5 ± 1.7	500	1.6 ± 2.0	0.463

*Significant at 0.05 level

## Discussion

The purpose of this study was not only to obtain the parameters for a single proximal humerus but also to use simple geometric parameters to describe the proximal humerus shape in the entire population of North China region based on measurements of multiple samples. Our results are expected to facilitate the increase in perceptions of the proximal humeral morphology among surgeons, assist with the design of shoulder prostheses and proximal humeral fixation instruments, and improve the clinical outcome of trauma and other disease of the proximal humerus.

Previously reported humeral parameters were mostly obtained from the cadaver specimens or X-ray based 2-dimensinal measurements. Several CT-dependent studies have been published as well in recent years. CT could eliminate the errors brought from posture and tube projection angles and CT could be easily acquired, stored and applied to reconstruct 3D models. Most studies based on CT measurements are from Western sources. This study, aimed to precisely establish the anatomical parameters dataset of the proximal humerus in a Northern Chinese population, differs from previous studies in population selection and measured parameters.

The morphometric parameters of each anatomical structure of the humeral head were measured in detail in this study. AAHH, RCHH, DHH and THH in cHHP and tHHP were used to describe the morphology of the humeral head; MO and PO were used to describe the relative position of the humeral head to the proximal humeral shaft; IA, AANS and RCNS were used to describe the morphology of metaphysis; superior, Inferior, anterior and CAHAN were used to describe the morphology of anatomical neck; HGT, HLT, VIG, LIG, DIG, CAIG,UAGT and LAGT were used to describe the morphology of proximal anterolateral region of the humerus. The morphology of the proximal humerus was converted into above parameters, and thus, the morphology characteristics of patient’s humerus could be communicated among doctors and researchers without pictures or other visual ways.

A total of 25 proximal humeral parameters were measured, 12 parameters were significantly larger in males than females (*P* < 0.05). There were apparently different physiological structures between the sexes, nearly all the differences in parameters between men and women were related to the size of proximal humeral anatomical landmarks such as the RCHH, HGT or VIG, rather than the parameters such as the IA, AANS or CAHAN; nevertheless, no difference was observed in RCNS and humeral head offset, either medially or posteriorly. These results suggests that the female humerus is not a simple scaled-down version of male humerus; therefore, sex differences should be considered when designing medical devices. In present study, left and right side of humerus showed strong symmetry. The contralateral humerus can serve as a reliable reference for injury side during the treatment.

The geometric parameters of intertubercular groove have not been paid much attention in previous studies. The long head of the bicep tendon passes through the intertubercular groove and covered by the transverse humeral ligament. The influence of anatomical and morphological variations of the intertubercular groove could be responsible for shoulder disorders such as subluxations, tears and tendinitis of biceps tendon. In addition to measuring the length, depth and concave angle of intertubercular groove, this study measured the volume of the intertubercular groove by mask filling simulation. This parameter that may better reflect the containment function of the intertubercular groove for more exploration of the correlation between the morphological variations and the long head of the bicep tendon disorders in the future.

We focused on the geometry of the lateral to the greater tuberosity of the humerus where the proximal humerus plate placement was often performed. Poor fit of plate placement could cause pain, limited mobility, screw loosening and even plate fracture. Parameters we measured could optimize the plate design, in order to increase the plate fitting and decrease mobility of unstable fracture segments for better surgical results.

Correlation analysis showed no correlation with age for all proximal humeral morphometric parameters. Proximal humeral morphology may not be susceptible to aging or daily use. Complex interrelationship exists among the dimensional parameters of the humeral head, suggested that the morphology of the humeral head existed mutually synergistic developmental mechanisms.

In previous studies, the humerus was usually simplified as a sphere, making measurement of the parameters convenient [8]. We described the humeral head morphology by using two orthogonal, osculating circles and measured the parameters of the humeral head in both two circles which could present the morphology in cHHP and tHHP plane of humeral head, for more accurate measurement results. Parameters measured from the same humeral head in sagittal versus coronal planes were compared using paired t tests. Statistically significant differences were observed in curvature (average curvature on coronal humeral head plane—average curvature on transversal humeral head plane = 1.55 mm, *P* < 0.001), diameter (average diameter on coronal humeral head plane—average diameter on transversal humeral head plane = 3.06 mm, *P* < 0.001) and thickness (average thickness on coronal humeral head plane—average thickness on transversal humeral head plane = 1.03 mm, *P* < 0.001) of the same humeral head in coronal humeral head plane and transversal humeral head plane, suggesting that humeral head could not be well fitted as a sphere. However, the three-dimensional analysis using CT scans neglected the thickness of the articular cartilage, and the present findings might have underestimated the parameters of humeral head.

We obtained several findings by comparing humeral head morphology data for cohorts of different local populations. East Asian cohorts have smaller humeral head sizes compared with Western cohorts. In the coronal plane of the humeral head, Eastern cohorts have smaller curvature and diameter of the humeral head compared with Western cohorts. However, the arc and thickness of the humeral head are greater in East Asians, indicating that there was more articular surface coverage of the humeral head on the coronal plane in East Asian cohorts. By analyzing the degree of dispersion of the two groups, larger standard deviations of all differential parameters were found in Western cohorts. This finding may indicate that a smaller range of prosthesis size was required in East Asian cohorts. Our data can be used as the humerus morphological parameters of the yellow race to provide a reference for subsequent research as well.

The shoulder prosthesis is a component that needs to be implanted and used for the long-term. Improving design accuracy and simulating the real situation are important to reduce unnecessary load and wear [20]. Having a digital understanding of proximal humeral morphology plays a role in diagnosing and classifying disease, designing prosthesis and instrument, enhancing surgical precision and guiding patient recovery.

## Data Availability

The datasets used and/analyzed during the current study are available from the corresponding author on reasonable request.

## Abbreviations

CT: Computed tomography
3D: Three-dimensional
MO: Medial offset of humeral head
PO: Posterior offset of humeral head
RCHH: Radius of curvature of humeral head
AAHH: Angle of arc of humeral head
DHH: Diameter of humeral head
THH: Thickness of humeral head
cHHP: Coronal humeral head plane
tHHP: Transversal humeral head plane
IA: Humeral head inclination angle
AAHN: Arc angle of humeral neck-shaft
RCHN: Radius of curvature of humeral neck-shaft
CAHAN: Concave angle of humeral anatomical neck
HGT: Height of greater tuberosity height
HLT: Height of lesser tuberosity height
DIG: Depth of intertubercular groove
CAIG: Concave angle of intertubercular groove
VIG: Volume of intertubercular groove
UAGT: Upper angle of greater tuberosity
LAGT: Lower angle of greater tuberosity
